# Inflammatory mediators profile in patients hospitalized with COVID-19: A comparative study

**DOI:** 10.3389/fimmu.2022.964179

**Published:** 2022-07-25

**Authors:** Abdisa Tufa, Tewodros Haile Gebremariam, Tsegahun Manyazewal, Tewodros Getinet, Dominic-Luc Webb, Per M. Hellström, Solomon Genet

**Affiliations:** ^1^ Department of Medical Biochemistry, School of Medicine, College of Health Sciences, Addis Ababa University, Addis Ababa, Ethiopia; ^2^ Department of Internal Medicine, School of Medicine, College of Health Sciences, Addis Ababa University, Addis Ababa, Ethiopia; ^3^ Centre for Innovative Drug Development and Therapeutic Trials for Africa (CDT-Africa), College of Health Sciences, Addis Ababa University, Addis Ababa, Ethiopia; ^4^ School of Public Health, Saint Paul’s Hospital Millennium Medical College, Addis Ababa, Ethiopia; ^5^ Gastroenterology and Hepatology Unit, Department of Medical Sciences, Uppsala University, Uppsala, Sweden

**Keywords:** SARS-CoV-2, COVID-19, cytokines, chemokine, immune response, inflammatory mediators, Ethiopia

## Abstract

Abnormal inflammatory mediator concentrations during SARS-CoV-2 infection may represent disease severity. We aimed to assess plasma inflammatory mediator concentrations in patients with SARS-CoV-2 in Addis Ababa, Ethiopia. In this study, 260 adults: 126 hospitalized patients with confirmed COVID-19 sorted into severity groups: severe (n=68) and mild or moderate (n=58), and 134 healthy controls were enrolled. We quantified 39 plasma inflammatory mediators using multiplex ELISA. Spearman rank correlation and Mann-Whitney U test were used to identify mechanistically coupled inflammatory mediators and compare disease severity. Compared to healthy controls, patients with COVID-19 had significantly higher levels of interleukins 1α, 2, 6, 7, 8, 10 and 15, C-reactive protein (CRP), serum amyloid A (SAA), intercellular adhesion molecule 1 (ICAM-1), vascular cell adhesion protein 1 (VCAM-1), IFN-γ-inducible protein-10 (IP-10, CXCL10), macrophage inflammatory protein-1 alpha (MIP-1α, CCL3), eotaxin-3 (CCL26), interferon-gamma (IFN-γ), tumor necrosis factor-α (TNF-α), basic fibroblast growth factor (bFGF), placental growth factor (PlGF), and fms-like tyrosine kinase 1 (Flt-1). Patients with severe COVID-19 had higher IL-10 and lower macrophage-derived chemokine (MDC, CCL22) compared to the mild or moderate group (*P<0.05*). In the receiver operating characteristic curve, SAA, IL-6 and CRP showed strong sensitivity and specificity in predicting the severity and prognosis of COVID-19. Greater age and higher CRP had a significant association with disease severity (*P<0.05*). Our findings reveal that CRP, SAA, VCAM-1, CXCL10, CCL22 and IL-10 levels are promising biomarkers for COVID-19 disease severity, suggesting that plasma inflammatory mediators could be used as warning indicators of COVID-19 severity, aid in COVID-19 prognosis and treatment.

## Introduction

SARS-CoV-2 has unusually potent transmission and toxicity. It shares severe flu-like symptoms and acute respiratory distress syndrome of zoonotic diseases with other SARS variations (e.g., SARS-CoV) and the Middle East Respiratory Syndrome (MERS) (1). Individuals who test positive for SARS-CoV-2 by molecular diagnostics, such as reverse-transcriptase polymerase chain reaction (RT-PCR), are initially classified as asymptomatic or pre-symptomatic. Furthermore, COVID-19 symptomatology is commonly related to fever, cough, dyspnea and myalgia or weariness (2). Sputum production, headaches, hemoptysis and diarrhea are all minor symptoms. Infectious pneumonia is the hallmark of severe disease and consequences may include acute respiratory distress syndrome (ARDS), abrupt heart damage and secondary infection (3). The severity of a disease is determined by its symptomatology. The mean incubation period for COVID-19 was 6 days globally, but around 7 days on the Chinese mainland, which will aid in determining the time of infection and making disease control decisions (4).

Many investigations have indicated that COVID-19 patients had increased levels of circulating proinflammatory cytokines (5). During the rapid course of COVID-19, a cytokine storm sometimes ensues, altering the immune system by lowering lymphocyte counts, particularly T cells (6). When the immune system as a whole is disrupted, immune cells release a huge number of pro-inflammatory cytokines and chemokines, which exacerbates the cytokine storm (7). In the most severe cases of COVID-19, IL-6 levels are greatly elevated, and this is one of the factors that leads to cytokine production (8). In COVID-19 patients, viral-induced hyper-inflammation is strongly linked to disease severity, and it is even the leading cause of mortality (9). As a result of an erroneous immune response, COVID-19’s critical and severe cases are characterized by sepsis and multiple organ failure (10).

Chemokines are important inflammatory mediators that help the immune system respond to infections; nonetheless, excessive production is the major cause of hyperinflammation (11). Significant levels of macrophage chemo-attractants such as CXCL10 and CCL2/MCP-1, as well as neutrophil chemo-attractants like CXCL2 and CXCL8, enhance immune cell migration to the site of infection, which is consistent with mononuclear cell infiltrates in COVID-19 patients’ lung tissues (12). After lysis, neutrophils, which play a role in early antiviral defense, are known to promote cytotoxic lung inflammation and high neutrophil-to-lymphocyte ratios have been linked to poor COVID-19 patient outcomes (13). According to a recent meta-analysis, chemokines (CCL2, CXCL10 and CCL11) were shown to be higher in severe COVID-19 patients than in moderate cases (14). CXCL10 and CCL2 are biomarkers that have been associated with disease severity and the risk of death in COVID-19 patients (15).

COVID-19 cases progressively increased in Ethiopia, with a total of 485,047 new cases and 7,525 deaths as of June 22nd, 2022. However, progression to severe diseases and rate of case-fatality have been substantially lower compared to countries in the northern part of the world. Dynamics of population aging, epidemiological interventions, and weather conditions may explain the phenomenon, while immune response against SARS-CoV-2 may influence disease severity that evidence is limited in Ethiopia.

Thus, this study aimed to assess the concentrations of circulating inflammatory mediators in patients hospitalized with COVID-19 in Ethiopia and investigate their contributions to the diagnosis and follow-up of disease severity.

## Materials and methods

### Study design and setting

A comparative cross-sectional study was conducted at Tikur Anbessa Specialized Hospital (TASH), the largest referral and teaching hospital in Ethiopia and led by the College of Health Sciences, Addis Ababa University. The hospital has 700 beds and serves over 500,000 patients in the outpatient department and 370,000-400,000 inpatients every year, admitting and treating patients for several medical specialties and subspecialties. It was one of the cores COVID-19 diagnosis and treatment centers in Ethiopia. Data for this study were collected from January 27, 2021, to December 30, 2021.

### Study participants

The study included 126 COVID-19 patients admitted to TASH with laboratory-confirmed and clinically diagnosed COVID-19 using a convenient sampling method and 134 healthy controls who had no contact with known COVID-19 suspected individuals for at least 14 days and had a negative RT-PCR. All participants were 18 years and older. Healthy control participants were selected from administrative staff and medical students at College of Health Sciences. Participants who were anemic, pregnant or had taken a corticosteroid or immunosuppressant within 14 days of admission were all excluded from the study.

The patients in this study did not get a SARS-CoV-2 vaccine and they had not had any antibiotics, anti-IL-6R, corticosteroids, statins, biological therapy prior to enrolling in the study or routine use of dexamethasone or remdesivir which may alter the relationship between inflammatory mediators and eventual outcomes.

### Operational definitions

Mild/moderate cases: mild are those which are not hypoxic and without evidence of pneumonia; moderate are with clinical signs of pneumonia (fever, cough) but no signs of severe pneumonia (oxygen saturation ≥ 90%).

Severe cases: admitted to intensive care unit due to severe hypoxia (oxygen saturation <90%) (16).

### Specimen collection

All patients came to the hospital within the first week of onset of symptoms for further diagnosis and treatment based on the standard of care. Of these, consecutive patients were evaluated for eligibility and enrolled in the study following their written consent. Relevant sociodemographic and health-related data were collected using a structured questionnaire.

Within 24 hours of their admission, 5 ml of venous blood sample was collected from each participant for measurement of inflammatory mediators. EDTA-plasma was separated from whole blood and immediately frozen at -80° C until assay. Following a signed sample transfer agreement approved by the national authority and following standard sample transportation procedures, specimens were sent to the Department of Medical Sciences, Gastroenterology and Hepatology Unit, Uppsala University, Sweden (17), for immunological assays. The same procedures were followed with the participants in the healthy control group, where 5 ml of blood was collected, and their sociodemographic and health-related data collected following their written consent.

### Inflammatory mediators analysis

A human cytokine 39-plex ELISA assay was used (Meso Scale Diagnostics, Rockville, MD, USA) to measure:

– basic fibroblast growth factor (bFGF);
– C- reactive protein (CRP);
– Eosinophil chemotactic proteins (CCL11, CCL26);
– Fms-like tyrosine kinase 1 (Flt-1);
– Granulocyte-macrophage colony-stimulating factor (GM-CSF);
– Intercellular adhesion molecule 1 (ICAM-1);
– Interferons-γ (IFN-γ);
– IFN-γ-inducible protein-10 (CXCL10);
– Interleukins (IL-1α, IL-1β, IL-2, IL-4, IL-5, IL-6, IL-7, IL-8, IL-10, IL-12p70, IL-12/IL-23p40, IL-13, IL-15, IL-16, IL-17A);
– Macrophage-derived chemokine (CCL22);
– Macrophage-inflammatory protein (CCL3, CCL4);
– Monocyte chemoattractant protein (CCL13), placental growth factor (PIGF);
– Serum amyloid A (SAA);
– Thymus activation-regulated chemokine (TARC, CCL17);
– Tumor necrosis factors (TNF-α, TNF-β);
– Tyrosine-protein kinase receptor (Tie-2);
– Vascular cell adhesion protein 1 (VCAM-1); and
– Vascular endothelial growth factor (VEGF-A, VEGF-C, VEGF-D)

Testings were performed at the Department of Medical Sciences, Gastroenterology and Hepatology Unit, Uppsala University, Sweden (17).

### Statistical analysis

IBM SPSS version 25.0 (Chicago, IL, USA) and Prism version 8 (San Diego, CA, USA) were used for data analysis. Descriptive summary measures including frequency, percentages, mean with standard deviation (SD) and median with interquartile range (IQR) were used to describe basic features of the study data. Chi-square test was used to determine associations between categorical variables. Shapiro-Wilk test was used to determine normality. A Bonferroni-adjusted Mann-Whitney U test was used to compare biomarker concentrations by group. Binary logistic regression was used to identify predictors of disease severity. Receiver operating characteristics (ROC) curve was employed to set cutoff points to predict disease status and disease severity, and the Spearman rank correlation was done to see the correlation between inflammatory mediators. *P-value < 0.05* was considered as statistically significant.

## Results

### Sociodemographic and clinical characteristics of participants

A total of 260 participants were included: 126 patients with COVID-19 and 134 healthy controls. Males made up 155 (60%) of the participants. Based on disease severity, the patients were categorized further into two groups: severe cases (68, 54%) and mild or moderate cases (58, 46%). There was a significant difference in the median age between the mild or moderate and severe cases [32 years (IQR 20-78) versus 60 years (IQR 22-86)] (**Table 1**).

**Table 1:** Demographics characteristic of COVID-19 patients and healthy controls.

	Healthy Controls,n=134	Casesn=126	Mild/Moderaten=58	Severe,n=68	Total,n=260	P-value
Age, Years, Median (IQR)	24 (20-55)	50 (20-86)	32 (20-78)	60 (22-86)		**<0.0001** ^a^, ^b^
Gender, Male,%	83 (61.94)	72 (57.14)	33 (56.89)	39 (57.35)	155 (59.6)	.431^a*^,.959^b*^

*Chi-square test.

a comparison between healthy controls and cases.

b comparison between mild or moderate and severe groups. Bold p value emphasizes 0.05 criteria met.

There were 64 (50.8%) COVID-19 patients with single or multiple co-morbidities such as diabetes (16%), hypertension (14%), cardiovascular disease (CVD) (17%), cancer (17%) and chronic lung disease (10%). Co-morbidities with diabetes and chronic lung disease were considerably higher in the severe than the mild or moderate group (**Table 2**).

**Table 2:** Comorbidity by COVID severity classification.

	All patients, n (%)	Mild/Moderate, n (%)	Severe, n (%)	P-value
**Any of following**				
Diabetes	20 (15.9)	5 (8.6)	15 (22.1)	**.040**
Hypertension	17 (13.5)	5 (8.6)	12 (17.6)	.139
CVD	21 (16.7)	10 (17.2)	11 (16.2)	.873
Cancer	21 (16.7)	12 (20.7)	9 (13.2)	.263
Chronic lung disease	12 (9.5)	2 (3.4)	10 (14.7)	**.032**
Comorbidity	64 (50.8)	27 (46.6)	37 (54.4)	
Non-comorbidity	62 (49.2)	31 (53.4)	31 (45.6)	.379
**Total patients (n)**	126	58	68	

Chi-square test was used. Bold p value emphasizes 0.05 criteria met. CVD, Chronic cardiovascular disease. Individuals have multiple comorbidity illness.

### Inflammatory mediator plasma concentrations

**Table 3** summarizes the concentrations of circulating inflammatory biomarkers in study samples. Compared to the healthy controls, patients with COVID-19 had significantly higher circulating concentrations of 19 inflammatory mediators, namely interleukins 1α, 2, 6, 7, 8, 10 and 15, CRP, SAA, ICAM-1, VCAM-1, CXCL10, CCL3, CCL26, IFN-γ, TNF-α, bFGF, PlGF and Flt-1 (*P < 0.05*, **Table 4**). This variation between the two groups persists even after age was adjusted, thus comparing those with the age < 40 years in the two groups (**Table 5**). However, IL-2, IL-13, CCL26, IFN-γ, and bFGF concentrations were not statistically significant following the Bonferroni adjustment for age.

**Table 3:** Concentration of inflammatory biomarkers sorted as healthy controls and COVID-19 patients.

Parameters	Healthy Controls, n=134, Median (IQR)	Cases, n=126, Median (IQR)	P-value
CRP (*mg/L)*	1 (0.40, 2.50)	82.50 (22.75,157.3)	<.0001*
ICAM-1(*mg/L)*	0.50 (0.40, 0.60)	0.88 (0.62,1.24)	<.0001*
SAA (*mg/L)*	1.90 (1.20, 3.70)	209.5 (46, 777.5)	<.001*
VCAM-1(*mg/L)*	0.6 (0.5, 0.7)	1.04 (0.78, 1.32)	<.001*
CCL11 (*ng/L)*	152.9 (88.67,244.6)	102.6 (68.25,148.8)	<.001*
CCL26 (*ng/L)*	22.06 (13.49,31.83)	26.76 (19.74,38.44)	.0002*
CXCL10 (*ng/L)*	145.1(96.92,196.0)	1582 (284.9, 3169)	<.001*
CCL2 (*ng/L)*	46.84 (34.50,73.88)	50.78 (37.17,92.48)	.0977
CCL13 (*ng/L)*	96.67 (61.27,138.5)	67.16 (50.86,103.0)	.0002*
CCL22 (*ng/L)*	725.7 (597.2,880.9)	438.9 (296.3,651.0)	<.0001*
CCL3 (*ng/L)*	7.32 (5.35,9.57)	13.46 (9.19,19.51)	<.0001*
CCL4 (*ng/L)*	69.94 (53.32,91.02)	61.38 (49.66,85.14)	.0917
CCL17 (*ng/L)*	185.4 (95.57,265.5)	70.77 (43.67,126.6)	<.0001*
IFN-Ƴ (*ng/L)*	3.17 (2.20,5.47)	4.95 (2.51,19.80)	<.001*
IL-10 (*ng/L)*	0.19 (0.15,0.26)	1.11 (0.40, 2.78)	<.0001*
IL-12p70 (*ng/L)*	0.20 (0.16,0.30)	0.21 (0.13,0.35)	.9185
IL-13 (*ng/L)*	0.85 (0.44,1.33)	0.99 (0.54, 1.51)	.0648
IL-1β (*ng/L)*	0.17 (0.11,0.24)	0.11 (0.00, 0.27)	**.0433**
IL-2 (*ng/L)*	0.53 (0.34,0.77)	0.66 (0.41,1.05)	**.0035**
IL-4 (*ng/L)*	0.03 (0.02,0.04)	0.04 (0.01, 0.06)	.3085
IL-6 (*ng/L)*	0.40 (0.28,0.62)	5.13 (1.93, 11.77)	<.0001*
IL-8 (*ng/L)*	6.81 (4.47,8.95)	14.11 (7.75, 27.03)	<.0001*
TNF-α (*ng/L)*	1.21 (0.98,1.49)	1.83 (1.19,2.69)	<.0001*
GM-CSF (*ng/L)*	0.00 (0.0,0.04)	0.03 (0.0,0.13)	<.0001*
IL-12/IL-23p40 (*ng/L)*	59.20 (47.28,76.98)	40.27 (12.99,76.30)	<.0001*
IL-15 (*ng/L)*	1.23 (1.05,1.42)	3.71 (2.15,5.20)	<.0001*
IL-16 (*ng/L)*	201.0 (136.7,534.4)	161.9 (113.8, 266.8)	.0011*
IL-17A (*ng/L)*	1.45 (1.02, 2.01)	1.61 (0.73, 2.79)	.7975
IL-1α (*ng/L)*	1.54 (0.94, 2.38)	2.56 (1.79, 3.89)	<.0001*
IL-5 (*ng/L)*	0.28 (0.11, 0.65)	0.17 (0.06, 0.53)	**.0207**
IL-7 (*ng/L)*	2.46 (1.76, 4.05)	4.30 (2.25, 7.54)	<.0001*
TNF-β (*ng/L)*	0.14 (0.10,0.19)	0.09 (0.05, 0.15)	<.0001*
VEGF (*ng/L)*	130.1 (60.12,247.6)	44.27 (8.59,180.0)	<.0001*
Flt-1(*ng/L)*	82.80 (66.23,99.21)	410.1 (170.7, 951.9)	<.0001*
PlGF (*ng/L)*	3.87 (3.34, 4.44)	8.77 (5.67,11.91)	<.0001*
Tie-2 (*ng/L)*	3265 (2662, 3797)	1970 (1659, 2398)	<.0001*
VEGF-C (*ng/L)*	204.1 (144.3, 336.2)	109.4 (44.78, 175.0)	<.0001*
VEGF-D (*ng/L)*	1343 (975.1, 1894)	1283 (793.3, 2125)	.8696
bFGF (*ng/L)*	5.19 (2.87, 16.99)	14.95 (5.58, 33.35)	<.0001*

Bold p value emphasizes 0.05 criteria met. *p value emphasizes Bonferroni (0.05/39) criteria met. Analytes were: Basic fibroblast growth factor, (bFGF); C-reactive protein, (CRP); eosinophil chemotactic proteins, (CCL11, CCL26); fms-like tyrosine kinase 1, (Flt-1); granulocyte-macrophage colony-stimulating factor, (GM-CSF); intercellular adhesion molecule 1, (ICAM-1); interferons, (IFN-γ); IFN-γ-inducible protein-10, (CXCL10); interleukins, (IL-1α, IL-1β, IL-2, IL-4, IL-5, IL-6, IL-7, IL-8, IL-10, IL-12p70, IL-12/IL-23p40, IL-13, IL-15, IL-16, IL-17A); macrophage-derived chemokine, (CCL22); macrophage-inflammatory protein (CCL3, CCL4); monocyte chemoattractant protein (CCL2, CCL13); placental growth factor (PIGF); serum amyloid A (SAA); thymus activation-regulated chemokine (CCL17); tumor necrosis factors (TNF-α, TNF-β); tyrosine-protein kinase receptor (Tie-2);vascular cell adhesion protein 1 (VCAM-1); vascular endothelial growth factor (VEGF-A, VEGF-C, VEGF-D). Samples were analyzed by a validated multiplex ELISA with intra- and inter-assay coefficient of variation typically below 10%.

**Table 4:** Concentration of inflammatory biomarkers in mild/moderate versus severe COVID-19 patients.

Parameters	Mild/Moderate, n=58, Median (IQR)	Severe, n=68, Median (IQR)	P-value
CRP (*mg/L)*	**44.50 (9.0, 110.3)**	**99.50 (43.25, 187.0)**	.0007*
ICAM-1(*mg/L)*	**0.74 (0.52, 1.14)**	**0.97 (0.67, 1.29)**	**.0082**
SAA (*mg/L)*	**111.0 (19.25,335.5)**	**422.0 (139.0,883.3)**	<.0001*
VCAM-1(*mg/L)*	**0.94 (0.72,1.22)**	**1.12 (0.86,1.37)**	**.0095**
CCL11 (*ng/L)*	114.7 (70.82,162.1)	93.41(67.61,133.5)	.1945
CCL26 (*ng/L)*	28.49 (19.03,37.54)	25.47 (20.41,39.60)	.9388
CXCL10 (*ng/L)*	**471.5 (168.8,1826)**	**2084 (721.1,4228)**	<.0001*
CCL2 (*ng/L)*	55.51 (41.01,97.44)	45.63 (35.55,87.95)	.1499
CCL13 (*ng/L)*	60.40 (48.32,90.30)	75.36 (53.26,111.6)	.1427
CCL22 (*ng/L)*	**563.0 (326.1,762.9)**	**380.4 (280.7,504.3)**	**.0036**
CCL3 (*ng/L)*	13.06 (9.36, 20.52)	13.54 (8.57,19.39)	.8348
CCL4 (*ng/L)*	61.01 (49.18, 81.78)	61.70 (50.14,86.63)	.9204
CCL17 (*ng/L)*	68.64 (45.04, 180.0)	71.94 (43.08,109.2)	.3889
IFN-Ƴ (*ng/L)*	5.99 (2.98, 23.11)	4.24 (1.87, 15.77)	.2639
IL-10 (*ng/L)*	**0.60 (0.31, 2.18)**	**1.49 (0.55, 3.78)**	**.0025**
IL-12p70 (*ng/L)*	0.21 (0.15, 0.34)	0.24 (0.12, 0.38)	.7706
IL-13 (*ng/L)*	1.14 (0.76, 1.70)	0.89 (0.50,1.43)	.0698
IL-1β (*ng/L)*	0.14 (0.0, 0.27)	0.060 (0.0, 0.25)	.6639
IL-2 (*ng/L)*	0.71 (0.36, 1.03)	0.61 (0.46, 1.08)	.4399
IL-4 (*ng/L)*	0.04 (0.02, 0.06)	0.03 (0.01,0.06)	.7863
IL-6 (*ng/L)*	4.78 (1.58, 11.21)	5.61 (2.55, 14.40)	.1715
IL-8 (*ng/L)*	13.83 (7.43, 26.27)	14.23 (8.24, 27.71)	.9504
TNF-α (*ng/L)*	1.71 (1.17, 2.81)	1.88 (1.22, 2.71)	.6076
GM-CSF (*ng/L)*	0.04 (0.0, 0.14)	0.01 (0.0, 0.10)	.4333
IL-12/IL-23p40 (*ng/L)*	**60.56 (28.58, 111.8)**	**29.90 (11.20, 52.28)**	.0005*
IL-15 (*ng/L)*	**2.66 (1.68, 5.01)**	**4.03 (3.09, 5.22)**	**.0037**
IL-16 (*ng/L)*	**138.5 (98.65, 247.8)**	**179.1 (129.1, 271.5)**	**.0098**
IL-17A (*ng/L)*	**2.34 (1.15, 3.47)**	**0.94 (0.50,2.18)**	.0001*
IL-1α (*ng/L)*	2.43 (1.88, 4.07)	2.68 (1.59, 3.87)	.9718
IL-5 (*ng/L)*	**0.33 (0.16, 0.76)**	**0.09 (0.0, 0.28)**	<.0001*
IL-7 (*ng/L)*	**2.71 (1.78, 5.27)**	**5.69 (3.49, 10.44)**	<.0001*
TNF-β (*ng/L)*	**0.13 (0.09,0.17)**	**0.06 (0.04, 0.13)**	<.0001*
VEGF (*ng/L)*	41.65 (7.83, 136.8)	48.95 (8.89, 191.6)	.5632
Flt-1 (*ng/L)*	**201.6 (107.8, 423.2)**	**701.6 (331.2, 1783)**	<.0001*
PlGF (*ng/L)*	8.62 (5.10, 10.97)	8.88 (6.25, 13.59)	.1669
Tie-2 (*ng/L)*	2091 (1680,2499)	1923 (1644, 2229)	.3442
VEGF-C (*ng/L)*	109.4 (43.50,191.0)	107.8 (46.16,169.2)	.8026
VEGF-D (*ng/L)*	**1061 (746.3,1563)**	**1686 (922.4,2384)**	**.0071**
bFGF (*ng/L)*	14.63 (4.43,34.33)	15.31 (8.44,32.84)	.5251

Bold p value emphasizes 0.05 criteria met. *p value emphasizes Bonferroni (0.05/39) criteria met.

**Table 5:** Concentrations of inflammatory biomarkers in healthy controls and cases groups for adjusted age (≤40 years).

Parameters	Healthy Controls, n=126, Median (IQR)	Cases, n=54, Median (IQR)	P-value
CRP (*mg/L)*	1(0.4, 2.4)	67.50 (12.5,115.3)	<.0001*
ICAM-1(*mg/L)*	0.5 (0.4, 0.6)	0.86 (0.5, 1.29)	<.0001*
SAA (*mg/L)*	1. 9 (1.2, 3.6)	131 (17, 508.8)	<.0001*
VCAM-1(*mg/L)*	0.6 (0.5, 0.7)	0.9 (0.7, 1.1)	<.0001*
CCL11 (*ng/L)*	153.8 (89.3,244.6)	90.7 (57.8,145.8)	<.0001*
CCL26 (*ng/L)*	21.6 (13.4, 32.2)	26.8 (20.8, 37.5)	**.0053**
CXCL10 (*ng/L)*	145.1 (96.9, 191.8)	463.4 (159.8,1944)	<.0001*
CCL2 (*ng/L)*	45.9 (34.5, 72.4)	45.6 (35.6, 86.5)	.6157
CCL13 (*ng/L)*	92.2 (59.8, 138.2)	60.6 (49.8, 92.8)	.0005*
CCL22 (*ng/L)*	723.7 (600.9,876.1)	501.1 (308.7,743)	<.0001*
CCL3 (*ng/L)*	7.4 (5.3, 9.6)	12.9 (9.1, 18.3)	<.0001*
CCL4 (*ng/L)*	69.9 (53.9, 87.6)	59.6 (48, 81.8)	**.0460**
CCL17 (*ng/L)*	187.7 (106.5, 265.5)	63.7 (41.6,152.9)	<.0001*
IFN-Ƴ (*ng/L)*	3.2 (2.2, 5.5)	4 (1.7, 19.1)	**.0376**
IL-10 (*ng/L)*	0.2 (0.1, 0.3)	0.8 (0.3, 2.7)	<.0001*
IL-12p70 (*ng/L)*	0.2 (0.2, 0.3)	0.2 (0.2, 0.4)	.3699
IL-13 (*ng/L)*	0.8 (0.4, 1.3)	1.1 (0.8,1.7)	**.0036**
IL-1β (*ng/L)*	0.2 (0.1, 0.2)	0.1 (0.0, 0.3)	.0974
IL-2 (*ng/L)*	0.5 (0.3, 0.8)	0.7 (0.5, 1.1)	**.0016**
IL-4 (*ng/L)*	0.03 (0.02, 0.04)	0.04 (0.02, 0.06)	.1804
IL-6 (*ng/L)*	0.4 (0.3, 0.6)	3.6 (1.8, 10.7)	<.0001*
IL-8 (*ng/L)*	6.8 (4.4, 9.3)	11.1 (6.1, 34.2)	<.0001*
TNF-α (*ng/L)*	1.2 (0.9, 1.5)	1.6 (1.1, 2.6)	.0012*
GM-CSF (*ng/L)*	0.0 (0.0, 0.04)	0.04 (0.0, 0.2)	.0002*
IL-12/IL-23p40 (*ng/L)*	59.2 (47.3, 77.2)	54.4 (13.4, 84.8)	.1528
IL-15 (*ng/L)*	1.2 (1, 1.4)	2.9 (1.8, 5.2)	<.0001*
IL-16 (*ng/L)*	200 (134.3, 590.3)	126.5 (94.3,202.7)	<.0001*
IL-17A (*ng/L)*	1.5 (1, 2)	1.9 (0.9, 3.4)	.2010
IL-1α (*ng/L)*	1.5 (0.9, 2.4)	2.5 (1.9, 3.5)	<.0001*
IL-5 (*ng/L)*	0.28 (0.1, 0.6)	0.26 (0.1, 0.7)	.7479
IL-7 (*ng/L)*	2.5 (1.8, 4.1)	3.3 (1.9, 5.8)	.0595
TNF-β (*ng/L)*	0.14 (0.1, 0.2)	0.12 (0.07, 0.16)	**.0283**
VEGF (*ng/L)*	127.6 (60.1, 238.1)	41.7 (12.4, 167.8)	<.0001*
Flt-1 (*ng/L)*	83.2 (66.1, 100.7)	214.2 (136.5,618.)	<.0001*
PlGF (*ng/L)*	3.9 (3.3, 4.4)	7.8 (4.9, 11.2)	<.0001*
Tie-2 (*ng/L)*	3284 (2683, 3845)	2098 (1731, 2549)	<.0001*
VEGF-C (*ng/L)*	206.5 (146.3,324.3)	113.5 (45.1,195.2)	<.0001*
VEGF-D (*ng/L)*	1335 (975.1,1882)	1493 (918.4, 2496)	.1389
bFGF (*ng/L)*	5.3 (2.9, 17.1)	12.6 (4.1, 31.3)	**.0208**

Bold p value emphasizes 0.05 criteria met. *p value emphasizes Bonferroni (0.05/39) criteria met.

Severe COVID-19 patients had significantly higher levels of CRP, ICAM-1, SAA, VCAM-1, CXCL10, IL-10, IL-15, IL-16, IL-7, Flt-1 and VEGF-D than mild or moderate COVID-19 patients. However, plasma levels of ICAM-1, VCAM-1, IL-10, IL-15, IL-16 and VEGF-D in the severe patients were comparable with mild/moderate groups after Bonferroni correction. CCL22, IL-12/IL-23p40, IL-5, IL-17A and TNF-β levels, on the other hand, were greater in mild or moderate COVID-19 patients than in severe COVID-19 patients (*P < 0.05*, **Table 4**). Between COVID-19 patients and healthy controls, median chemokine concentrations (CCL2 and CCL4) were not statistically significant (*P > 0.05*, **Table 3**). Based on the correlations of 30 significantly elevated inflammatory mediators; CRP, SAA, IL-6, IL-8, CXCL10, TNF-α, IL-10, VEGF-D, CCL22 and VCAM-1 were the ones that discriminated between mild or moderate and severe cases (**Table 6**).

**Table 6:** Correlations of inflammatory mediators. Data from all subjects (healthy controls, mild/moderate and severe cases).

Parameters		CRPn=260	SAAn=260	IL-6n=260	IL-8n=260	CXCL10n=260	TNFαn=260	IL-10n=260	VEGF-Dn=260	CCL22n=260	VCAM-1n=260
**CRP**	R-value P-value		0.923 0.000	0.846 0.000	0.497 0.000	0.453 0.000	0.464 0.000	0.655 0.000	-0.059 0.343	-0.455 0.000	0.622 0.000
**SAA**	R-value P-value	0.923 0.000		0.797 0.000	0.442 0.000	0.707 0.000	0.441 0.000	0.656 0.000	-0.059 0.340	-0.458 0.000	0.610 0.000
**IL-6**	R-value P-value	0.846 0.000	0.797 0.000		0.506 0.000	0.636 0.000	0.479 0.000	0.706 0.000	0.010 0.868	-0.437 3.730E-014	0.630 0.000
**IL-8**	R-value P-value	0.497 0.000	0.442 0.000	0.506 0.000		0.453 0.000	0.413 4.593E-012	0.434 1.033E-013	-0.013 0.843	-0.292 0.000	0.407 1.132E-011
**CXCL10**	R-value P-value	0.453 0.000	0.707 0.000	0.636 0.00	0.453 0.000		0.624 0.000	0.679 0.000	-0.208 0.001	-0.343 0.000	0.614 0.000
**TNF-α**	R-value P-value	0.464 0.000	0.441 0.000	0.479 0.000	0.413 4.593E-012	0.624 0.000		0.518 0.000	-0.243 0.000	-0.154 0.013	0.587 0.000
**IL-10**	R-value P-value	0.655 0.000	0.656 0.000	0.706 0.000	0.434 1.033E-013	0.679 0.000	0.518 0.000		-0.105 0.092	-0.438 1.799E-014	0.575 0.000
**VEGF-D**	R-value P-value	-0.059 0.343	-0.059 0.34	0.010 0.868	-0.013 0.843	-0.208 0.001	-0.243 0.000	-0.105 0.092		0.032 0.605	-0.011 0.854
**CCL22**	R-value P-value	-0.455 0.000	-0.458 0.000	-0.437 3.730E-014	-0.292 0.000	-0.343 0.000	-0.154 0.013	-0.438 1.799E-014	0.032 0.605		-0.375 0.000
**VCAM-1**	R-value P-value	0.622 0.000	0.610 0.000	0.630 0.000	0.407 1.132E-011	0.614 0.000	0.587 0.000	0.575 0.000	-0.011 0.854	0.375 0.000	

The pair(s) of variables with positive correlation coefficients and P values below 0.05 tend to increase together. For the pairs with negative correlation coefficients and P values below 0.05, one variable tends to decrease while the other increases. For pairs with P values greater than 0.05, there is no significant linear relationship between the two variables.

**Figure 1:**
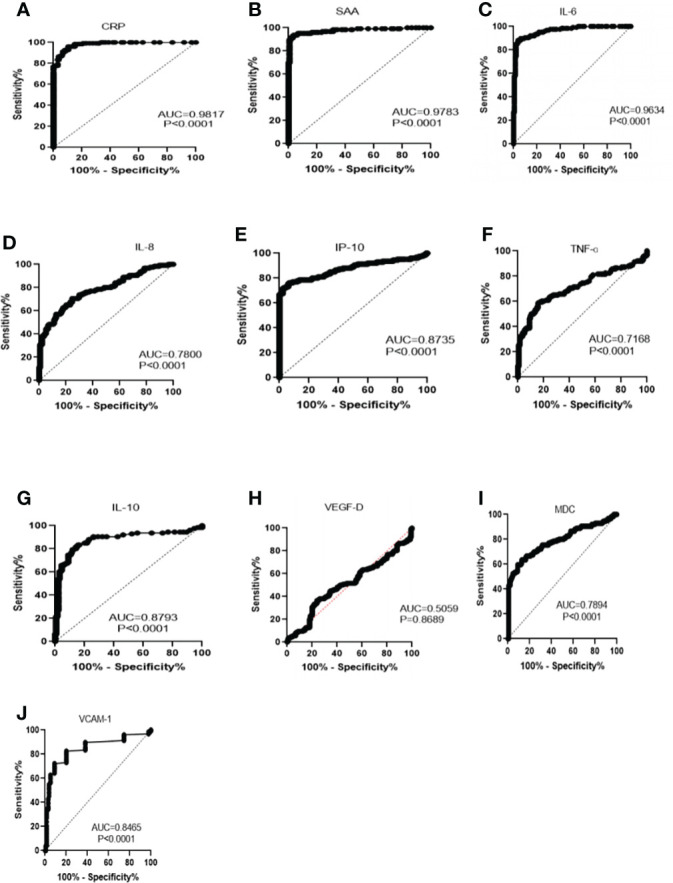
Receiver operating characteristics analysis of healthy control subjects versus COVID-19 patients, using specific biomarkers **(A)** CRP, C-reactive protein. **(B)** SAA, serum amyloid A **(C)** IL-6, interleukin 6. **(D)** IL-8, interleukin 8. **(E)** IP-10, interferon-γ-inducible protein-10. **(F)** TNF-α, tumor necrosis- α. **(G)** IL-10, interleukin-10. **(H)** VEGF-D, vascular endothelial growth factor-D. **(I)** MDC, macrophage-derived chemokine (MDC). **(J)** VCAM-1, vascular cell adhesion protein 1.

**Figure 2:**
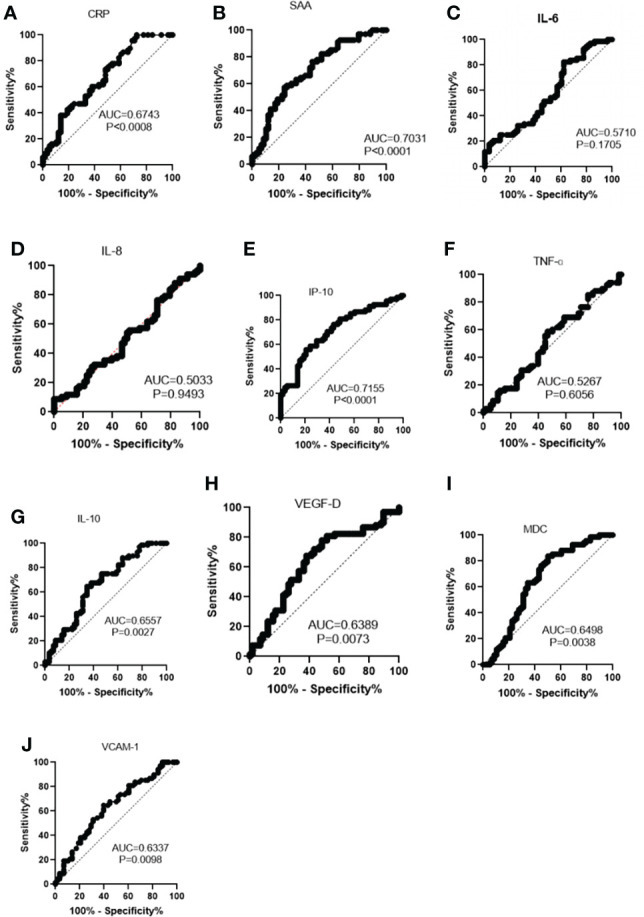
Receiver operating characteristics curve of indicators between mild/moderate and severe COVID-19 for the prediction of severity. **(A)** CRP, C-reactive protein. **(B)** SAA, serum amyloid A **(C)** IL-6, interleukin 6. **(D)** IL-8, interleukin 8. **(E)** IP-10, interferon-γ-inducible protein-10. **(F)** TNF-α, tumor necrosis- α. **(G)** IL-10, interleukin-10. **(H)** VEGF-D, vascular endothelial growth factor-D. **(I)** MDC, macrophage-derived chemokine (MDC). **(J)** VCAM-1, vascular cell adhesion protein 1.

The diagnostic value of inflammatory mediators for illness severity was assessed using the receiver operating characteristic (ROC) curve and the area under the ROC curve (AUC) (**Table 7** and **Figure 1**). AUC (95 percent confidence intervals) were CRP 0.9817 (0.9703-0.9931), SAA 0.9783 (0.9610-0.9956), IL-6 0.9634 (0.9425-0.9843), IL-8 0.7800 (0.7238-0.8362), CXCL10 0.8735 (0.8265-0.9204), TNF-α 0.7168 (0.6521-0.7816), IL-10 0.8793 (0.8328-0.9257), VEGF-D 0.5059 (0.4347-0.5772), CCL22 0.7894 (0.7324-0.8463) and VCAM-1 0.8465 (0.7960- 0.8971) respectively (*P < 0.05*). SAA, CRP and IL-6 have very high sensitivity and specificity. TNF-α and VEGF-D have poor sensitivity and specificity when compared to CRP, SAA and IL-6. The hierarchy among the 10 selected inflammatory mediators in distinguishing between healthy controls and COVID-19 patients, as well as between mild or moderate and severe COVID-19 patients, was validated by ROC and AUC analyses (**Tables 7** and **8**). **Figure 2** demonstrates the use of a ROC curve to distinguish between mild/moderate and severe COVID-19 cases for severity prediction.

**Table 7:** Area under the receiver operating characteristic curve, sensitivity, specificity & cutoff values of specific inflammatory markers of distinguishing healthy controls and cases.

Variable	AUC (95% CI)	Sensitivity % (95% CI)	Specificity% (95% CI)	Cutoff	P-value
CRP	0.9817(0.9703-0.9931)	75.4(67.2-82.1)	100(97.2- 100)	> 22.50	**<.0001**
SAA	0.9783(0.9610-0.9956)	88.9(82.2- 93.3)	99.3(95.9-99.9)	> 11.50	**<.0001**
IL-6	0.9634(0.9425-0.9843)	82.5(74.9- 88.2)	97.8(93.6- 99.4)	> 1.49	**<.0001**
IL-8	0.7800(0.7238-0.8362)	38.1(30.1- 46.8)	97.8(93.6- 99.4)	> 18.92	**<.0001**
CXCL10	0.8735(0.8265-0.9204)	69.8(61.3- 77.2)	97.8(93.6- 99.4)	> 374.2	**<.0001**
TNF-α	0.7168(0.6521-0.7816)	30.9(23.5- 39.5)	97.8(93.6- 99.4)	> 2.505	**<.0001**
IL-10	0.8793(0.8328- 0.9257)	53.2(44.5- 61.7)	97.8(93.6- 99.4)	> 1.010	**<.0001**
VEGF-D	0.5059(0.4347- 0.5772)	3.9(1.7- 8.9)	97.8(93.6- 99.4)	< 429.9	.8689
CCL22	0.7894(0.7324- 0.8463)	46.8(38.3- 55.5)	97(92.6 - 98.8)	< 418.8	**<.0001**
VCAM-1	0.8465(0.7960- 0.8971)	32.5(24.9- 41.1)	98.5(94.7- 99.7)	> 1.215	**<.0001**

Bold p value emphasizes 0.05 criteria met. Cut off value units for CRP, SAA and VCAM-1 were (mg/L) and IL-6, IL-8, CXCL10, TNF-α, IL-10, VEGF-D and CCL22 were (ng/L).

**Table 8:** Area under the receiver operating characteristic curve, sensitivity, specificity & cutoff values of specific markers in discriminating mild/moderate and severe COVID-19 patients.

Variable	AUC (95% CI)	Sensitivity % (95% CI)	Specificity% (95% CI)	Cutoff	P-value
CRP	0.6743(0.5799- 0.7687)	7.4(3.2 - 16.1)	98.3(90.9-99.9)	> 395	**.0008**
SAA	0.7031(0.6115 -0.7947)	5.9(2.3- 14.2)	98.3(90.9- 99.9)	> 1074	**<.0001**
IL-6	0.5710(0.4696 -0.6724)	11.8(6.1- 21.5)	100(93.8- 100)	> 77.72	.1705
IL-8	0.5033(0.4015- 0.6051)	7.4(3.2- 16.1)	100(93.8 - 100)	> 305.7	.9493
CXCL10	0.7155(0.6263- 0.8047)	20.6(12.7- 31.6)	98.3(90.9- 99.9)	> 5185	**<.0001**
TNF-α	0.5267(0.4246 -0.6289)	1.5(0.1- 7.9)	98.3(90.9- 99.9)	> 36.32	.6056
IL-10	0.6557(0.5589- 0.7524)	2.9(0.5 - 10.1)	100(93.8- 100)	> 46.46	**.0027**
VEGF-D	0.6389(0.5404-0.7375)	7.4(3.2 -16.1)	96.6(88.3- 99.4)	< 482.3	**.0073**
CCL22	0.6498(0.5495-0.7502)	4.4(1.2- 12.2)	93.1(83.6- 97.3)	< 156.1	**.0038**
VCAM-1	0.6337(0.5364 -0.7311)	2.9(0.5- 10.1)	98.3(90.9- 99.9)	> 2.120	**.0098**

Bold p value emphasizes 0.05 criteria met. Cut off value units for CRP, SAA and VCAM-1 were (mg/L) and IL-6, IL-8, CXCL10, TNF-α, IL-10, VEGF-D and CCL22 were (ng/L).

**Table 9** shows a binary logistic regression analysis that links CRP and age to COVID-19 disease severity. These findings indicated that CRP and age were statistically significant (*P < 0.05*). The correlation between inflammatory mediator levels and mortality in severe patients was determined. The inflammatory mediators that correlated with mortality were ICAM-1, CRP, CCL26, IL-6, IL-8, IL-10, IL-15, VEGF, CXCL10, CCL2, CCL3, CCL4, FIt1 and TNF α (**Supplementary Table 1**).

**Table 9:** Binary logistic regression analysis indicating factors associated with disease severity.

Variable	AOR (95% CI)	P-value
Age	1.056 (1.033-1.081)	<0.001
CRP	1.004(1.000-1.008)	0.044

AOR=Adjusted odds ratio; co-morbidity and gender were insignificant with p-value (0.696 and 0.730).

## Discussion

This study assessed the concentrations of 39 circulating inflammatory mediators in patients with SARS-CoV-2, using healthy comparators, and investigated the contributions of inflammatory mediators on the diagnosis and follow-up of disease severity. The findings showed that compared to healthy controls, patients with COVID-19 had significantly higher levels of interleukins 1α, 2, 6, 7, 8, 10 and 15, CRP, SAA, ICAM-1, VCAM-1, CXCL10, CCL3, CCL26, IFN-γ, TNF-α, bFGF, PlGF, and Flt-1. Patients with severe COVID-19 had higher IL-10 and lower CCL22 compared to the mild or moderate group. SAA, IL-6, and CRP showed strong sensitivity and specificity in predicting the severity and prognosis of COVID-19. Greater age and higher CRP had a significant association with disease severity. CRP, SAA, VCAM-1, CXCL10, CCL22 and IL-10 levels were promising biomarkers for COVID-19 disease severity. This study is the first comparative study of COVID-19 patients in Ethiopia, to the best of our knowledge, to investigate different inflammatory mediators profiles in discriminating between healthy controls and COVID-19 patients; mild/moderate and severe cases and prognosis, classification and predicting disease severity. SARS-CoV-2 infection can cause varied inflammatory response abnormalities. The pathogenesis of many viral infections, including coronaviruses, is connected to aberrant virus-induced immune responses (18). Immune responses, particularly expression of inflammatory mediators, are closely regulated in normal physiology; deviations from this control might result in different biochemical and clinical manifestations (19).

The average age of the severe group was much higher than mild/moderate group, in line with earlier research (20). This showed that age could be a risk factor for COVID-19 severity, and it was assumed that ACE2 density was shown to be positively connected with age (21, 22). Gender distribution in the group was not statistically significant. However, male patients were more likely to develop severe illness in the severe group than in the other groups. This could be due to gender-linked immunological differences (23).

Pro-inflammatory cytokines and interferons (IFNs) were shown to be greater in COVID-19 patients (24). These cytokines aid in the elimination of infections as well as the maintenance of cellular homeostasis. Dysregulated pro-inflammatory cytokine release, on the other hand, contributes to cytokine storm, a potentially fatal condition caused by inflammatory cells’ excessive cytokine production (25). Inflammatory mediator levels were found to be significantly higher in patients than in the healthy controls group. This study is consistent with a prior study that compared patients with severe COVID-19 to those with mild disease (26). In comparison to mild/moderate cases of COVID-19, plasma from patients with severe cases showed a greater tendency for levels of IL-6, IL-8 and TNF-α indicating a pro-inflammatory response. This research supports the findings of the prior research (27).

CRP is increased in response to IL-6, IL-1β and TNF-α activation during infection and systemic inflammation (28). CRP levels were significantly greater in the severe group compared to the other groups in this study, which is consistent with previous studies that reported CRP levels as potential markers for disease severity (29, 30). CRP is a non-specific measure used to distinguish between infectious and non-infectious diseases caused by viruses or bacteria (31, 32). Many studies suggest that CRP could be used to predict prognosis even before the onset of sickness as a biomarker for COVID-19 severity and that an increase in CRP is linked to a poor COVID-19 prognosis (30). It can also be used as an early indicator of infection and inflammation. The current study and previous studies demonstrated that increased levels of CRP have clinical diagnostic and prognostic value during COVID-19 infection.

Endothelial cells aid in the recruitment of leukocytes from the circulation to infection and inflammatory sites (33). Inflammatory cytokines initiate different kinase cascades during SARS-CoV-2 infection, leading to the activation of transcription factors like NF-κB, which in turn stimulate adhesion molecules like ICAM-1 and VCAM-1. ICAM-1 promotes leukocyte arrest and firm adhesion, as well as monocyte and lymphocyte transmigration. In the current study, COVID-19 patients had higher levels of ICAM-1 and VCAM-1 than healthy control and the severe group had higher levels than the mild or moderate group, which was consistent with earlier findings (34). This suggests that ICAM-1 and VCAM-1 could be used as a biomarker to predict the severity of COVID-19 disease and may have a role in coagulation dysfunction.

In line with the previous study, SAA levels in the current study were considerably higher in the COVID-19 patients than in healthy controls. SAA is released by hepatocyte cells in response to inflammatory cytokines such as IL-6, TNF-α and IL-1β during an acute phase response. One of the most common clinical symptoms of severe COVID-19 is ARDS (35). There were patients with ARDS who had a considerably higher level of SAA. SAA could be used as a biomarker to track the evolution of respiratory disorders like COVID-19, according to our findings. The median levels of both CRP and SAA were greater in COVID-19 patients than in healthy controls after age adjustment (comparing age < 40 years in both COVID-19 patients and healthy controls). CRP and SAA were both linked to disease severity; have a strong and positive linear correlation. Since measurement of different inflammatory mediators is time-consuming and costly, correlation analysis aids clinical prediction of one biomarker in terms of another. Thus, correlations were performed in the current investigation, implying that plasma levels of CRP, SAA, IL-6, CXCL10, VCAM-1, and IL-10, among the linked, exhibit a significant and beneficial association with COVID-19 patient progression. CRP, SAA, IL-6, CXCL10, VCAM-1, TNF-α and IL-10 all had a negative relationship with plasma levels of VEGF-D and CCL22.

Many researchers have attempted to explore the expression of pro-inflammatory chemokines linked to SARS-CoV-2 infection in both *in-vitro* and *in-vivo* models in order to better understand the mechanism by which SARS-CoV-2 causes harmful lung inflammation. In this study, in individuals with COVID-19, CXCL10 levels were higher than in healthy controls and the same is true after age adjustment. Furthermore, when the viral infection was linked with a pulmonary pathology (e.g., influenza and SARS-CoV-2), CXCL10 levels were higher than when the infection was associated with a non-pulmonary pathology (e.g., human rhinovirus) (15). CXCL10 levels were significantly greater in severe SARS-CoV-2-positive patients than in mild or moderate patients, implying that CXCL10 could be a useful biomarker for distinguishing between mild/moderate and severe COVID-19 patients and predicting disease severity. CCL2 and CCL11 levels in the mild/moderate and severe groups were comparable. Monocyte migration from the bloodstream through the vascular endothelium mediated by CCL2 is required for normal immunological monitoring in tissues in response to inflammation (36). CCL2 expression increases significantly during the early acute phase of infection and then gradually diminishes as the disease progresses (37). As a result, early monitoring of CCL2 and CCL11 levels and responding on any elevations could be a potential method for preventing COVID-19 from advancing from mild to severe. However, one meta-analysis found that severe cases of COVID-19 had greater levels of chemokines (CCL2 and CCL11) than moderate ones (14). This discrepancy might be due to a difference in the sample collection time between our study and the studies included in the meta-analysis.

In SARS, Middle East respiratory syndrome-CoV and SARS-CoV-2 infected patients, excessive cytokine production has been associated to pulmonary inflammation and acute lung damage (18, 38). Anti-inflammatory cytokine IL-10 was shown to be more strongly related with disease progression and severe acute kidney injury than the proinflammatory cytokines IL-6 and IL-8 in COVID-19 patients with severe sickness (39). IL-10 is a cytokine with many functions that reduces the inflammatory response by direct effect on macrophages and T and B-cells (40). It is known to produce T-cell anergy or non-responsiveness in anti-tumour cell responses as well as viral infection (41). There are theories that IL-10 has both an anti-inflammatory (classical role) and a pro-inflammatory effect (non-classical role). The rapid increase in IL-10 could be viewed as an effort to control tissue damage and hyperinflammation given its well-established roles as an anti-inflammatory and immunosuppressive cytokine. However, the increase in IL-10 in severe cases of COVID-19 in this investigation could be due to IL-10’s pro-inflammatory activity. This is consistent with a prior study that found that increased IL-10 levels may have a proinflammatory and immune-activating role in COVID-19 progression (42, 43). Furthermore, as compared to healthy controls and mild/moderate COVID-19 patients, plasma IL-7 levels were considerably higher in severe COVID-19 patients in this study. This research supports the findings of the prior research (44). T-cell growth and functions are dependent on IL-7. Increased levels of IL-7 in the blood have been linked to a reduction in the number of T cells in the body (45). As a feedback response to lymphopenia, serum IL-7 concentration was also negatively related to the number of T cells, CD4^+^ and CD8^+^ cells, highlighting the link between lymphopenia and higher IL-7 levels in SARS-CoV-2 infection (46).

In this study, mild/moderate COVID-19 cases had considerably greater levels of IL-12/IL-23p40 than severe COVID-19 patients and healthy controls. This is in line with the findings of the prior study (47). Induction of IL-12 is essential to sustain NK cell counts during the early stages of SARS-CoV-2 infection and this induction could aid in evasion from virus spreading. The number of peripheral NK cells in patients with severe COVID-19 was much lower than in healthy people (48). Furthermore, IL-17A is a homeostatic proinflammatory cytokine that is also involved in the development of autoimmune disorders. Our findings revealed that IL-17A levels in severe COVID-19 patients were significantly lower than in mild or moderate COVID-19 patients. A meta-analysis comparing IL-17A levels in severe and moderate patients found that severe patients had greater levels (49). The discrepancy could be due to the data limitations that were revealed during the meta-analysis.

Similarly, TGF-β levels were found to be decreased in severe patients compared to mild or moderate patients and healthy controls. This is consistent with the concept that serum TGF-β levels peak during the first two weeks of acute COVID-19 infection and limit NK cell function in a TGF-dependent way (50). As a result, premature TGF-β production is thought to be a characteristic of severe COVID-19. TGF-β is released from a variety of sources in response to SARS-CoV-2 infection, including dysregulated coagulation and fibrinolytic pathways; neutrophils infiltrating the lungs in large numbers and macrophages migrating to the lungs to phagocytize apoptotic bronchial epithelial cells, pneumocytes, T-lymphocytes and neutrophils (51). The potential of this cytokine to recruit more neutrophils and remodel the airways through modulating processes used by the virus to generate pulmonary fibrosis explains its influence in SARS-CoV-2 infection.

The severe group had significantly greater levels of VEGF-D than the mild or moderate group. There was a data shortage for this biomarker, but it was consistent with the prior research, which compared critical and severe group (52). We hypothesized that a high level of VEGF-D is linked to a storm of blood clots in COVID-19 patients, which eventually leads to disease severity.

The ROC curve data further revealed that CRP, SAA and IL-6 levels had excellent sensitivity and specificity for COVID-19 severity. This biomarker’s likelihood ratio value also accurately predicted illness dynamics, making it easier to recognize and act with underlying severe patients earlier, which was critical for lowering mortality. Binary logistic regression analysis found odds ratio (OR=1.056) for age, implying that the chance of being severe increases by 1.056 times with each unit rise in age. As people become older, their chance of becoming severely ill will increase. The same is true for CRP, with an OR of 1.004; a one-unit CRP increase has 1.004 times increased likelihood of being severe. Increases in age and CRP will enhance the likelihood of severity, according to the regression model. These data suggest that both increments of CRP and age have a role in the advancement of COVID-19 disease severity. The correlations of inflammatory mediator levels with patient’s survival status were seen. Few inflammatory mediators were found to be significantly linked with mortality. These relationships, however, tended to be weaker. IL-6 showed the highest association (r =.384, *p =0.001*) of all the variables. CRP levels and plasma levels of IL-6 had a strong correlation (r = 0.846; *P<0.05*) (**Table 6**). A crucial regulator of CRP synthesis is IL-6. In this study, there was a substantial association between the expression of these two inflammatory mediators and mortality.

This study has some limitations. It was difficult to make age-adjusted comparisons for elders as there were no such participants in the healthy controls group. The study did not make a further follow-up with the patients to draw a second round of blood specimens and monitor disease progression.

In conclusion, characterizing immune response is crucial for guiding public health interventions and measures. Thirty different inflammatory mediators levels were shown to be elevated as a result of the severity of the disease in this investigation, suggesting that they could be utilized to predict the severity and prognosis of COVID-19 patients. Among elevated plasma inflammatory mediator levels; CRP, ICAM-1, SAA, VCAM-1, CXCL10, IL-7, IL-10 and Flt-1 may suggest the severity of COVID-19. Increment of these inflammatory mediators in COVID-19 patients indicate an abnormal immune response and the emergence of a cytokine storm, which worsens the disease and may lead to death. As a result, the critical biomarkers determined in this study may aid in clinical care monitoring and COVID-19 treatment follow up.

## Data availability statement

The raw data supporting the conclusions of this article will be made available by the authors, without undue reservation.

## Ethics statement

The study was approved by the Ethiopian National Research Ethics Review Committee (NRERC) of the Ministry of Science and Higher Education, Ethiopia (Ref.No.MoSHE/04/246/837/21) and the Institutional Review Board of the College of Health Sciences, Addis Ababa University, Ethiopia (Protocol 004/21/Biochem). Permission to conduct the study was also obtained from TASH. All participants in the study (both COVID-19 patients and healthy controls) signed a written informed consent form.

## Author contributions

Credit roles were as follows: AT: design, preparation, data curation, data analysis, investigation, and methodology; roles/writing - original draft: writing - review and editing and correction; SG: design, conceptualization, formal analysis, investigation, methodology, project administration, resources, software, visualization, and roles/writing - review and editing; THG: clinical investigation, methodology, supervision, and writing - review and editing; TM: funding acquisition, methodology, project administration, resources, supervision, writing - review and editing; TG: performed statistics and produced tables and figures. PH: conceptualization, funding acquisition, investigation, methodology, project administration, resources, supervision, writing - review and editing. D-LW: conceptualization, data curation, formal analysis, funding acquisition, investigation, methodology, project administration, resources, software, supervision, validation, visualization, roles/writing - original draft, and writing - review and editing. AT, SG, THG, TM, TG, PH, and D-LW conceived the idea of the presented work and gave final approval of the version to be published. Specific contributions: AT wrote ethics approvals and recruited subjects. PH and D-LW funded clinical chemistry. All authors took part in study design. AT and D-LW performed clinical chemistry and analyzed data. AT and TG performed all statistics and produced tables and figures. AT and D-LW worked on initial draft. All authors took part in finalizing the paper. All authors contributed to the article and approved the submitted version.

## Funding

Centre for Innovative Drug Development and Therapeutic Trials for Africa (CDT-Africa), College of Health Sciences, Addis Ababa University, Addis Ababa supported expenses associated with data and sample collection. PH and D-LW, Gastroenterology and Hepatology Unit, Department of Medical Sciences, Uppsala University, Sweden support all expenses related to laboratory analysis.

## Acknowledgments

AT sincerely acknowledges support from CDT-Africa of Addis Ababa University and PH and D-LW, Department of Medical Sciences, Gastroenterology and Hepatology Unit, Uppsala University, Sweden. D-LW gratefully acknowledges support from Selander’s Foundation (2020 & 2021) as well as OE and Edla Johansson’s Science Foundation (2017 & 2018). D-LW and PH gratefully acknowledge funding from ALF-medel (Uppsala-Örebro region, ALF-899261) and Vetenskapsrådet (2017–02243).

## Conflict of interest

The authors declare that the research was conducted in the absence of any commercial or financial relationships that could be construed as a potential conflict of interest.

## Publisher’s note

All claims expressed in this article are solely those of the authors and do not necessarily represent those of their affiliated organizations, or those of the publisher, the editors and the reviewers. Any product that may be evaluated in this article, or claim that may be made by its manufacturer, is not guaranteed or endorsed by the publisher.
