# Supplementary material for: Inflammatory mediators profile in patients hospitalized with COVID-19: A comparative study
Source: Front Immunol. 2022 Jul 25;13:964179. doi: 10.3389/fimmu.2022.964179 (PMC9359079; doi:10.3389/fimmu.2022.964179)
Supplement: Supplementary file 1 [file Table_1.docx]

**SUPPLEMENTARY TABLE1**: Correlations between inflammatory mediator concentrations and outcome in severe patients.

| **Inflammatory mediators** | | **Mortality** |
| --- | --- | --- |
| ICAM-1 | R-value  P-Value | .379  **.001** |
| CRP | R-value  P-Value | .329  **.000** |
| SAA | R-value  P-Value | .120  .330 |
| VCAM-1 | R-value  P-Value | .208  .089 |
| CCL11 | R-value  P-Value | .100  .417 |
| CCL26 | R-value  P-Value | .329  **.006** |
| IL-8 | R-value  P-Value | .300  **.013** |
| CXCL10 | R-value  P-Value | .251  **.039** |
| CCL2 | R-value  P-Value | .393  **.001** |
| CCL13 | R-value  P-Value | -.012  .920 |
| CCL22 | R-value  P-Value | -.018  .884 |
| CCL3 | R-value  P-Value | .241  **.048** |
| CCL4 | R-value  P-Value | .256  **.035** |
| CCL17 | R-value  P-Value | -.054  .660 |
| IFN-γ | R-value  P-Value | .079  .522 |
| IL-10 | R-value  P-Value | .314  **.009** |
| IL-12p70 | R-value  P-Value | .072  .562 |
| IL-13 | R-value  P-Value | -.005  .969 |
| IL-1β | R-value  P-Value | .147  .232 |
| IL-2 | R-value  P-Value | .134  .275 |
| IL-4 | R-value  P-Value | .077  .534 |
| IL-6 | R-value  P-Value | .384  **.001** |
| TNF-α | R-value  P-Value | .308  **.011** |
| GM-CSF | R-value  P-Value | .135  .274 |
| IL-23p40 | R-value  P-Value | .060  .627 |
| IL-15 | R-value  P-Value | .380  **.001** |
| IL-16 | R-value  P-Value | .228  .062 |
| IL-17A | R-value  P-Value | .072  .557 |
| IL-1α | R-value  P-Value | .026  .835 |
| IL-5 | R-value  P-Value | .057  .646 |
| IL-7 | R-value  P-Value | -.106  .391 |
| TNF-β | R-value  P-Value | -.218  .074 |
| FIt1 | R-value  P-Value | .247  **.043** |
| PlGF | R-value  P-Value | .140  .255 |
| Tie-2 | R-value  P-Value | .111  .370 |
| VEGF | R-value  P-Value | -.359  **.003** |
| VEGF-C | R-value  P-Value | -.142  .248 |
| VEGF-D | R-value  P-Value | -.134  .275 |
| bFGF | R-value  P-Value | .123  .318 |

Bold p value emphasizes 0.05 criteria met.
